# Estimating the transfer function from neuronal activity to BOLD using simultaneous EEG-fMRI

**DOI:** 10.1016/j.neuroimage.2009.09.011

**Published:** 2010-01-15

**Authors:** M.J. Rosa, J. Kilner, F. Blankenburg, O. Josephs, W. Penny

**Affiliations:** a Wellcome Trust Centre for Neuroimaging, UCL Institute of Neurology, University College London, 12 Queen Square, WC1N 3BG, UK; b Department of Neurology and Bernstein Center for Computational Neuroscience, Charite, Berlin, Germany

## Abstract

Previous studies using combined electrical and hemodynamic measurements of brain activity, such as EEG and (BOLD) fMRI, have yielded discrepant results regarding the relationship between neuronal activity and the associated BOLD response. In particular, some studies suggest that this link, or transfer function, depends on the frequency content of neuronal activity, while others suggest that total neuronal power accounts for the changes in BOLD. Here we explored this dependency by comparing different frequency-dependent and -independent transfer functions, using simultaneous EEG-fMRI. Our results suggest that changes in BOLD are indeed associated with changes in the spectral profile of neuronal activity and that these changes do not arise from one specific spectral band. Instead they result from the dynamics of the various frequency components together, in particular, from the relative power between high and low frequencies. Understanding the nature of the link between neuronal activity and BOLD plays a crucial role in improving the interpretability of BOLD images as well as on the design of more robust and realistic models for the integration of EEG and fMRI.

## Introduction

Functional magnetic resonance imaging (fMRI), with blood oxygen level dependent (BOLD) contrast, is an established method for making inferences about regionally specific activations in the brain (Frackowiak et al., 2003). However, the relationship between BOLD and neuronal activity is still under debate, in particular, it is still unclear how the hemodynamic response is influenced by the temporal dynamics of the underlying neuronal activity.

One of the approaches used to study this relationship is to combine information from hemodynamic measures, such as fMRI, and electrophysiological measures, such as electroencephalography (EEG) and magnetoencephalography (MEG). EEG and MEG are well established non-invasive techniques and are well suited to studying the temporal dynamics of neuronal activity since they provide direct measurement of this activity with high temporal resolution (Hämäläinen et al., 1993).

In humans, the study of correlations between EEG and fMRI signals has been pioneered by epilepsy researchers, such as Lemieux et al. (2001) and Salek-Haddadi et al. (2002). However, most of our present knowledge about neurovascular coupling has come from animal research and the combination of metabolic/vascular measurements, such as cerebral blood flow (CBF), with local field potentials (LFPs) and single/multi-unit activity (S/MUA) recordings. LFPs correspond primarily to weighted averages of synchronized dendro-somatic components of synaptic signals in a neuronal population, while S/MUA measure the action potentials of a single neuronal cell or population of cells, respectively (Logothetis, 2008). These studies confirm that BOLD is indeed related to neuronal activity (Lauritzen, 2001), and although both LFPs and MUA correlate with the BOLD response, this response can be predicted more accurately from the LFPs (Logothetis et al., 2001).

More recently, Thomsen et al. (2004) and Viswanathan and Freeman (2007) have used co-localised measures of LFPs, MUA, and CBF in animals to show that when synaptic and spiking activity is uncoupled, changes in CBF do not reflect the underlying spiking activity and relate closer to the measured LFPs. These studies have therefore confirmed that BOLD primarily reflects changes in the synaptic input of neuronal populations as opposed to their spiking output. This reflects an emerging consensus in which BOLD is thought to result from pre-synaptic activity and the release of neurotransmitters, in particular glutamate (Friston, 2008). This release triggers a response in surrounding glial cells, especially astrocytes, leading to the generation of vasodilatory signals and consequently BOLD (Nair, 2005). As well as indirectly causing BOLD, glutamate will increase post-synaptic activity and therefore the LFP. Increases in LFP frequency would therefore be accompanied by faster glutamate recycling and consequently a larger BOLD signal.

While the above physiological perspective would suggest that BOLD should be sensitive to the frequency content of neuronal activity, results from the neuroimaging literature are not completely clear cut. For example, some studies (see next paragraph) suggest that BOLD is mainly dependent on the total energy, or total spectral power, of neuronal activity. Others (see next but one paragraph), suggest that BOLD is sensitive to a certain range of frequencies or results from more complicated dynamics.

Among those proposing a relationship between BOLD and total neuronal power, Wan et al. (2006) have found significant correlation between the mean power (mean square current source density estimates during visual stimulation) of source-reconstructed EEG data in human primary visual cortex and a neuronal efficacy parameter, derived from fitting a balloon model to fMRI data (Friston et al., 2000). Similarly, Nangini et al. (2008) propose that the energy density, as measured by the square of the equivalent current dipole (ECD) waveforms from source-reconstructed MEG data, is a better representation for the neuronal input functions than the stimulus functions conventionally used in convolution models for the analysis of fMRI data (Friston et al., 1995b). In addition to these studies, theoretical models for integrating EEG/MEG and fMRI (Nunez and Silberstein, 2000; Trujillo-Barreto et al., 2001; Babajani and Soltanian-Zadeh, 2006) assume a relationship between indices of neuronal activity and BOLD that is independent of the frequency of this activity. For instance, Babajani and Soltanian-Zadeh (2006) use a neural mass model of neuronal activity and propose that the squared post-synaptic membrane potential from both excitatory and inhibitory cells from a given cortical area drives increases in cerebral blood flow, and consequently BOLD.

Among those proposing a relation between BOLD and the frequency structure of electrophysiological signals, Goldman et al. (2002), Moosmann et al. (2003), and Laufs et al. (2003) have shown that reductions in ongoing scalp EEG alpha power in humans correlate with increases in BOLD activity. Lachaux et al. (2007) have found, using intra-cranial recordings in epileptic patients, a close spatial correspondence between regions of fMRI activation and sites showing EEG energy variation in the gamma band. Mukamel et al. (2005) have found positive correlations between LFPs and BOLD at high gamma-range frequencies ([40, 130] Hz) and negative correlations at low/alpha-range frequencies ([5, 15] Hz) in auditory cortex of neurosurgical patients. In addition, Niessing et al. (2005) have shown that fluctuations in hemodynamic response tightly correlate with the power of LFP oscillations, recorded in cat primary visual cortex, in the same high-frequency (gamma) range.

Kilner et al. (2005) note that from the perspective of fMRI, neuronal activation is proportional to relative metabolic demands or rate of energy dissipation (1/s units). In terms of EEG, the effect of activation is to shift the spectral profile toward higher frequencies (1/s units) with a reduction in amplitude. This led Kilner et al. (2005) to propose a ‘Heuristic’ model that links these two observations via a dimensionality analysis. This Heuristic specifies that BOLD activations are accompanied by an increase in the ‘average’ frequency of EEG neuronal activity, where average is defined in the root mean square (RMS) sense. Thus increases in higher frequencies, such as the gamma range, relative to lower frequencies, such as the alpha range, would lead to increases in BOLD. Conversely, increases in alpha relative to gamma would lead to decreases in BOLD.

Moreover, using data from Niessing et al. (2005) the Heuristic model has been shown to provide a better fit than a model based on gamma correlation alone (Kilner et al., 2007). In similar spirit to the idea underlying the Heuristic, Laufs et al. (2006a) have found that BOLD deactivations in humans are associated with increases in the ratio between theta and alpha bands (measured with scalp EEG), and that these deactivations cease when there is a decrease in this ratio and an increase in the beta/alpha ratio.

More recently, Goense and Logothetis (2008) used simultaneous intra-cortical LFP-BOLD recordings and a multiple regression model in which activity in many different frequency bands, covering the entire LFP range of frequencies, were used to predict BOLD activity in alert behaving monkeys. The results showed that all bands explained a significant part of the BOLD response.

The link between neuronal activity and BOLD has been investigated at both a microscopic scale, using invasive, co-localised recordings in animals (e. g. Logothetis et al., 2001; Niessing et al., 2005; Goense and Logothetis, 2008) and at a macroscopic scale using simultaneous EEG-fMRI in humans (Lemieux et al., 2001; Goldman et al., 2002; Laufs et al., 2003; Moosmann et al., 2003). A problem with the macroscopic approach is that the electrophysiological measure, EEG, is not co-localised with BOLD. This issue can be addressed by the use of principal component analysis (PCA) (Laufs et al., 2006b), independent component analysis (Eichele et al., 2005, 2009), or source reconstruction (Wan et al., 2006).

In this paper, we use simultaneous EEG-fMRI in humans and employ a visual flicker stimulation paradigm to elicit evoked activity in sensory cortex. As scalp EEG measures the activity of multiple distributed neuronal processes, we used a PCA approach to isolate activity that was primarily related to the stimulus paradigm. The resulting time series was then used as a surrogate for neuronal activity.

We then regressed the fMRI data onto convolved features of the power spectrum of the first principal component of the EEG data. We use a standard statistical parametric mapping (SPM) approach employing *F*-tests to compare models embodying different transfer functions. These are (i) a total power model, (ii) a frequency response model, comprising multiple regression onto power in different frequency bands, and (iii) a Heuristic model in which BOLD is predicted by the RMS EEG frequency.

The paper is structured as follows. In the Materials and methods section, we describe the experimental paradigm and the simultaneous acquisition of EEG and fMRI data. We also describe the pre-processing steps used for artefact removal and define the transfer functions investigated and the methods used to compare models. The Results section presents the results from the SPM analysis, and in the Discussion section these results are discussed in light of previous results from the literature.

## Materials and methods

### Subjects and task

Three healthy volunteers (three male, mean age = 35 ± 4 years) participated in the study after giving informed consent. Subjects were exposed to visual flicker stimuli of a number of different frequencies. A reversing black and white checkerboard (11 × 11 squares, size 13 cm × 13 cm) was delivered via a computer monitor (60 Hz refresh rate) and projected on a screen positioned 47 ± 1 cm from a 45° mirror located 11 ± 3 cm from the subject (visual angle = 6.5 ± 0.5°). The stimulation (reversing) frequencies used were 2.00, 3.75, 5.00, 6.00, 7.50, 10.00, 15.00, and 30.00 Hz. Stimuli were delivered in epochs of 5 scans (15.3 s), followed by periods of 15.3 s of rest (blank screen), and the order of stimulus blocks (e.g. 10 Hz, 6 Hz, 5 Hz, etc.) was randomised. Subjects were instructed to view a fixation cross which was visible during both rest and stimulus periods, and no overt response was required in either condition. Three consecutive sessions of the same experimental task were recorded for each subject. Although luminance levels were not held constant for the different flicker frequencies, the variations in luminance were measured using a lux meter placed in front of the visual display unit. This allowed luminance variations to be regressed out during subsequent statistical analyses, when required.

As the aim of our experiment was to investigate the neurovascular coupling driven by a large electrophysiological response in sensory cortex, the inter-subject variability was expected (and found) to be low. It is therefore appropriate (Penny and Holmes, 2006) to acquire data from a small number of subjects (three), to report results in the form of case studies, and to summarize these results using fixed-effects SPMs (see below). This follows the precedent of Wan et al. (2006) who also used a case study approach with a small number of subjects (five).

### EEG acquisition

EEG was acquired simultaneously with fMRI using a synchronized imaging protocol (Mandelkow et al., 2006) and an MR-compatible BrainAmp amplifier and BrainCap EEG cap with ring Ag/AgCl electrodes (Brainproducts GmbH, Munich, Germany). Raw EEG was sampled at 5 kHz and a low-pass filter (cutoff frequency: 1 kHz) was used. This system provided 29 EEG channels, 2 EOG channels, and 1 ECG channel. The electrodes were distributed according to the 10/20 system, and the reference electrode was located between Fz and Cz. EEG was also recorded outside of the MRI environment (in a dark and acoustically isolated room), so that the effect of MRI-induced artefacts and their removal could be assessed. We additionally measured the pulse using a pulse oxymeter attached to the subject's finger and the locations of the EEG electrodes were digitised with a Polhemus digitiser.

### fMRI acquisition

Images were acquired from a 1.5-T whole-body scanner (Magnetom Sonata, Siemens Medical, Erlangen, Germany) operated with its standard body transmit and CP head receive coil. The manufacturer's standard automatic 3D-shim procedure was performed at the beginning of each experiment. The scanner produced T2⁎-weighted images with a single-shot gradient-echo EPI sequence. Whole-brain images consisting of 34 contiguous transverse slices, on a 64 × 64 grid, were acquired every 3.06 s resulting in a total of 320 functional scans for each of the three sessions of each subject (slice thickness = 2 mm, gap between slices = 1 mm, repetition time TR = 90 ms, flip angle = 90°, echo time TE = 50 ms, field of view FOV = 192 × 192 mm^2^, and therefore 3 × 3 × 3 mm voxel resolution). Whole-brain structural scans were also acquired using a T1-weighted 3D-Modified Driven Equilibrium Fourier Transform (MDEFT) sequence (Deichmann et al., 2004) in 176 sagittal partitions with an image matrix of 256 × 256 (TR = 12 ms, TE = 4 ms, flip angle = 23°, and voxel size 1 × 1 × 1 mm).

### EEG data analysis

Acquisition of EEG in the MRI environment induces gradient and cardiac-related artefacts, such as the ballistocardiogram artefact (Goldman et al., 2000). The data acquired inside the scanner were corrected off-line using facilities in the Brain Vision Analyzer software package (Brainproducts GmbH, Munich, Germany) (Allen et al., 2000). First, the gradient artefact was removed via mean subtraction with template drift compensation. Cardiac-related artefacts were then removed by subtracting the first three principal components that were time locked to pulse oxymeter readings. EEG data acquired outside the scanner were not processed in this way. Both the data acquired inside and outside the scanner were then high-pass filtered (0.5 Hz) to reduce slow drifts in the signal.

After MR-related artefact removal and filtering, the inside and outside EEG data were visually inspected for other artefacts, such as eye-blinks, as well as movement-related artefacts. Due to their proximity to the subjects' eyes, the Fp1 and Fp2 electrodes contained too many eye-blink artefacts to be included in the analysis.

After visual inspection, the EEG data from the remaining channels were then processed to form a single representative ‘scalp EEG’ time series, by projecting the data onto a subspace defined by its first principal eigenvector *u*_1_.

In previous work, Moosmann et al. (2003) and Laufs et al. (2003) have generated a single representative time series by computing the mean over a subset of activated electrodes (e.g. 01, 02, P1, P2). We have used a spatial eigen decomposition method because this data-driven approach produces the single time series which, out of all possible linear projections, captures most variance in the original data. However, as brain activity in our paradigm is primarily driven by activity in visual cortex this spatial eigenmode is primarily loaded onto posterior electrodes, as is shown below.

The principal eigenvectors can be computed from a singular value decomposition (SVD) of the data. If *Y* is an *n*_*e*_ × *n*_*t*_ matrix of EEG data, with *n*_*e*_ electrodes and *n*_*t*_ time points, then an SVD gives *Y* = *USV*^*T*^, and the projection is given by $\tilde{y}=u_{1}^{T}Y$, where *u*_1_ is the first column of *U*.

To investigate the spectral properties of the scalp signal, $\tilde{y}(t)$, we decomposed it into the time-frequency domain. This decomposition was obtained by convolving the signal with Morlet wavelets, *G*, where for each time point *t* and frequency *f*:

(1) $$G(f,t)=A\exp(-t^{2}/2\sigma_{t}^{2})\exp(2i\pi ft),$$

where $A={\left(\sigma_{t}\sqrt{\pi }\right)}^{-1/2}$, *σ*_*t*_ = 1/(2π*σ*_*f*_), *σ*_*f*_ = *f*/*R*, and *R* = 7 is the ‘wavelet factor’. The time-varying power of the signal around frequency, *f*, is then given by the squared modulus of the convolution (Tallon-Baudry and Bertrand, 1999):

(2) $$P(f,t)={\left|G(f,t)*\tilde{y}(t)\right|}^{2},$$

and the power spectrum for all frequencies and time points can be represented by the matrix *P* with dimensions *n*_*f*_ × *n*_*t*_, where *n*_*f*_ is the number of frequencies.

### Transfer functions

From the spectrum of the EEG data, *P*, we constructed regressors defining the different transfer functions we were interested in comparing. These represent the functional link between neuronal activity and BOLD.

The first model, motivated by the result of Wan et al. (2006), assumes that neurovascular coupling is a power transducer. To this end we derived a feature corresponding to the ‘Total Power’ (TP) in the scalp EEG time series. This was obtained by summing the EEG power over all frequencies analyzed ([1, 40] Hz):

(3) $$q_{TP}(t)=\sum_{f=1}^{n_{f}}P(f,t).$$

The second model, following Goense and Logothetis (2008), assumes that BOLD is best explained by a linear combination of activity in different frequency bands. We refer to this as the ‘Frequency Response’ (FR) model and consider three variants, each with a different number of frequency bands. These comprise (i) three bands of low frequencies [1, 7] Hz, alpha frequencies [8, 15] Hz, and higher frequencies [5, 40] Hz; (ii) five bands of delta [1, 4] Hz, theta [4, 8] Hz, alpha [8, 13] Hz, beta [13, 30] Hz, and lower gamma [30, 40] Hz activity; and (iii) eight bands of 5 Hz each, from 1 to 40 Hz. The time series for each band were obtained by summing the power in the corresponding frequency interval, *b *= [*f*_min_, *f*_max_]:

(4) $$q_{\text{FR}}{\left(t\right)}_{b}=\sum_{f=f_{\text{min}}}^{f_{\text{max}}}P(f,t).$$

The resulting time series for each band, *b*, correspond to different columns of the same design matrix (see below).

The third model, which we refer to as the ‘Heuristic’ model based on Kilner et al. (2005), assumes that BOLD is best explained by a linear convolution of the ‘root mean squared frequency’ (RMSF) function. This is given by

(5) $$q_{\text{RMSF}}(t)=\sqrt{\sum_{f=1}^{n_{f}}f^{2}\tilde{P}(f,t)},$$

where $\tilde{P}$ is the corresponding normalised power spectrum of the representative scalp time series. This function describes how changes in the relative power of the different frequencies in the EEG spectrum could be associated with changes in BOLD.

We also investigated two variants of the Heuristic. The first, uses the un-normalised power spectrum *P*, instead of $\tilde{P}$:

(6) $$q_{\text{uRMSF}}(t)=\sqrt{\sum_{f=1}^{n_{f}}f^{2}P(f,t)}.$$

We refer to this as the ‘un-normalised Heuristic’ (u-Heuristic). Second, to test for the importance of the non-linearity introduced by the square root in the RMSF function, we defined the function,

(7) $$q_{\text{MSF}}(t)=\sum_{f=1}^{n_{f}}f^{2}\tilde{P}(f,t),$$

which is a linear version of Eq. (5). We refer to this as the ‘linear Heuristic’ (l-Heuristic) model.

To further test the importance of the non-linearity, we defined another function based on a linear convolution of the ‘mean frequency’ (MF) of the EEG signal:

(8) $$q_{\text{MF}}(t)=\sum_{f=1}^{n_{f}}f\tilde{P}(f,t).$$

Finally, we constructed one last frequency-independent transfer function purely based on variations of amplitude in the EEG signal, as captured by the global field power (GFP). The GFP corresponds to the root mean square deviations between all electrodes in a given potential field (Skrandies, 1995):

(9) $$q_{\text{GFP}}(t)=\sqrt{\sum_{i=1}^{n_{e}}{\left(U_{i}(t)-\overset{―}{U}(t)\right)}^{2}},$$

where $\overset{―}{U}(t)=\frac{1}{n_{e}}\sum_{j=1}^{n_{e}}U_{j}(t)$ is the mean of the potential across electrodes at a given time point. This is a reference-free measure and allowed us to compare the previously described transfer functions, which are all based on the power spectrum of the EEG data, with a measure based simply on the amplitude of the EEG signal.

For each of the above models, the time series were convolved with an informed basis set to accommodate variability in the hemodynamic response. This basis set includes the canonical hemodynamic response function (HRF), as well as its first temporal and dispersion derivatives (Friston et al., 1995a). The two derivative regressors allow for variations, across subjects and across the brain, in the peak response time and duration of the hemodynamic response. The temporal derivative, for example, allows for peak responses that are approximately one second earlier or later than is usual.

The convolved time series were then downsampled to match the fMRI sampling rate and served as regressors of interest in the subsequent general linear model (GLM) (Friston et al., 1995b).

As we are using an informed basis set with 3 basis functions, the Total Power, Heuristic, u-Heuristic, l-Heuristic, MF, and GFP models are implemented using 3 design matrix columns. There are therefore 3 corresponding regression coefficients of interest to estimate for each of these models. The Frequency Response model is implemented with 9, 15, or 24 columns for the 3-, 5-, or 8-band model, respectively. The coefficients of interest as well as the total number of parameters estimated for each function are summarised in Table 1.

### fMRI data analysis

The fMRI data were pre-processed with SPM8 software (http://www.fil.ion.ucl.ac.uk/spm/) implemented in Matlab (The Mathworks, Inc.). The first five scans of each session were discarded, and the pre-processing steps included (a) realigning the images to the first scan and coregistering the structural scan of each subject with the mean functional image from all sessions; (b) correcting for differences in acquisition time between slices and normalising all the functional and structural scans to a standard EPI template based on the Montreal Neurological Institute (MNI) reference brain in Talairach space (Talairach and Tournoux, 1988); and (c) smoothing the functional images (Gaussian kernel, 8 mm half width). The movement parameters obtained from the realignment step were included in the subsequent GLM analyses as confounding covariates (Table 1). The data were also high-pass filtered, with a cutoff period of 128 s.

We report analyses based on the first 100 scans of each session due to suspected movement-related (i.e. high amplitude and high-frequency) artefacts present in the EEG signal, after approximately 5 min of recording, in more than one session and subject. However, we later visually re-inspected the EEG signal and decided to include some of the previously discarded scans, and re-analysed the data using 200 scans per session. This new analysis yielded very similar results and strengthened the findings obtained with less data (see below).

For each subject, we first looked at the effect of the experimental task. We used the onsets of the stimuli as regressors, and inferences based on the statistical parametric maps (SPMs) from a fixed-effects group analysis were considered significant at *p* < 0.05, corrected for multiple comparisons using random field theory (Friston et al., 1995b). Inference was based on *F*-tests, which test for the additional variance explained by a set of regressors of interest. We also used these maps to generate a mask image, which we refer to as the ‘BOLD activation mask’. This mask allows us to look at correlations between model predictors and BOLD, limited to the voxels activated by the checkerboard stimuli.

### Model comparisons

In this section, we describe the comparisons between transfer functions that were performed in order to investigate the link between neuronal activity and BOLD.

We began by looking at correlations between individual functions and the BOLD signal, by using these functions in *separate design matrices*. This was followed by a more formal comparison, which included regressors from multiple models in the *same design matrix*.

Inference in both cases was based on *F*-tests. In the first case we test for the effect of each model alone, i.e. without taking into account the rest of the models. This is to reproduce previously published results, that each feature of neuronal activity is predictive of BOLD. In the second case, a significant *F*-statistic for a particular transfer function suggests that model explains BOLD variability that cannot be explained by any of the other functions in that design matrix (Friston et al., 1995b). This allows us to infer that one model is better than another.

These tests were performed using contrast vectors (Friston et al., 2007) that select the regressors of interest for each model, including the temporal and dispersion derivative regressors (Table 1). The criteria used to evaluate the models included the *F*-scores, the number of voxels above the *p* < 0.05 (FWE corrected) and *p* < 0.001 (uncorrected) thresholds, as well as the location of these voxels (inside or outside the ‘BOLD activation mask’) for each function (Table 3).

The transfer functions were compared as follows (a summary of these comparisons can be found in Table 1):

i. In order to ascertain whether our main transfer functions showed significant correlations with BOLD, as suggested by the results from the literature on which these functions were based (see Transfer functions section), we correlated the Total Power, Heuristic, and Frequency Response (3 bands) models individually with BOLD, as described above.
ii. Subsequently, we compared the frequency-dependent functions (Heuristic and Frequency Response) with the main frequency-independent function, Total Power. We implemented two pairwise comparisons (a) Total Power versus Heuristic and (b) Total Power versus Frequency Response (3 bands), which allowed us to probe whether the link between BOLD and neuronal activity is frequency dependent.
iii. We then implemented a three-way comparison (Total Power, Frequency Response with 3, 5 or 8 bands and Heuristic) to finally determine which transfer function provides a better fit to the BOLD data, when all models are taken into account.
iv. We also performed a similar comparison but we have included the GFP transfer function together with the previous models. This allowed us to assess whether a model based on the amplitude of the EEG signal, rather than its spectral content, was a better predictor of BOLD.
v. To determine whether the Frequency Response model performs better with less frequency bands, in particular with just a single band, we have performed two pairwise comparisons between (a) the Heuristic and the power in the Alpha frequencies (8 to 15 Hz) and between (b) the Heuristic and the power in the high (Beta/Gamma) frequency band (15 to 40 Hz).
vi. Finally, to investigate different properties of the Heuristic model, as described above, we implemented several pairwise comparisons. These included the Heuristic versus (a) the u-Heuristic, (b) the l-Heuristic, (c) the MF function, and (d) the Frequency Response model with 5 and 8 bands constructed using the normalised power spectrum.

For each of the above comparisons, we used a fixed-effects group analysis using 3-sessions of data from three subjects (9 sessions in total), giving rise to a total of 900 scans. Subsequent analyses based on 1800 scans (200 scans per session, as mentioned above) produced very similar results. These fixed-effects SPMs summarise the results over the three subjects (Penny and Holmes, 2006). We also computed SPMs for each subject in isolation, in a case study approach (see below).

The total number of regressors for each of the design matrices used is summarised in Table 1. For example, for the main three-way comparison (iii.) the design matrix employed 198 regressors (198 = 3 regressors of interest for Total Power, 3 for the Heuristic, 9 for the three-band Frequency Response model, 6 for the movement regressors, and 1 for the session mean × 9 sessions). The stimulus onset-based regressors were not included in these design matrices since they do not provide a plausible biological model, or link, between BOLD and underlying neuronal activity.

## Results

### Artefact correction and SVD

**Fig. 1:**
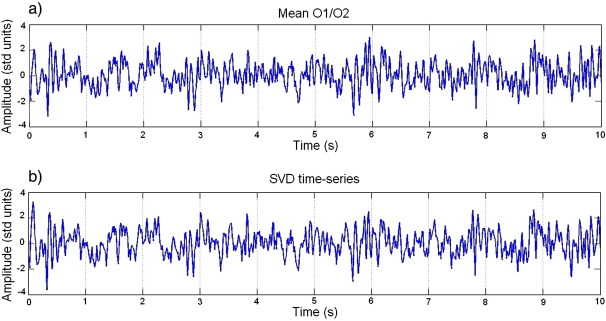
Example of artefact-corrected EEG time series for the first 10 seconds of the first visual stimulation period: (a) mean activity of electrodes O1 and O2. (b) Projection onto first principal component (SVD time series).

To remove scanning artefacts from the EEG, the data were processed as described in the Materials and methods section. Fig. 1 shows the first 10 s of an example time series from corrected EEG data for (a) the mean of electrodes O1 and O2 and (b) the scalp signal obtained from the SVD. As can be seen, the data appear uncontaminated by MR-related artefacts and is relatively free from other artefacts, such as eye-blinks. A prominent ∼ 10 Hz waveform can also be easily detected in these signals.

**Fig. 2:**
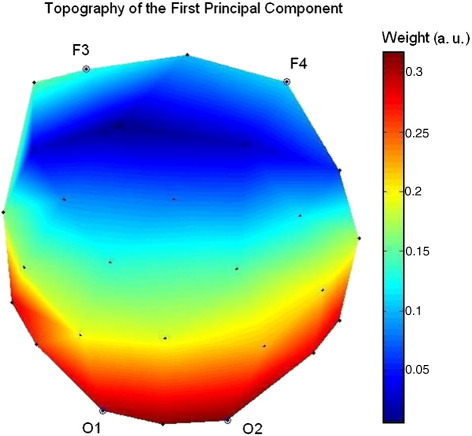
Topography (2D) of the EEG first principal component for a representative subject. The locations of the occipital and frontal electrodes are indicated by their respective names.

The fact that the time courses of these two signals look very similar (Fig. 1) was expected, since the first principal component of the EEG is primarily driven by activity from posterior regions. This is confirmed by plotting the topography of this component, as shown in Fig. 2. In addition, the first component explains 67% of the total variance of the data, which should provide a good representation of EEG activity.

**Fig. 3:**
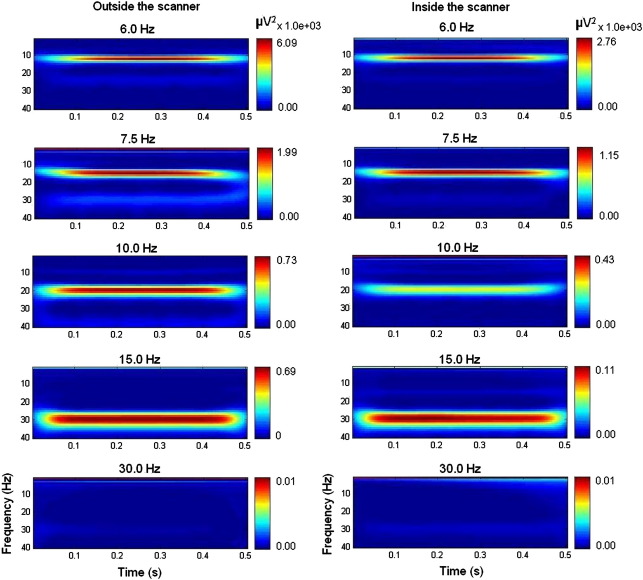
Power spectra of the SSVERs for EEG acquired outside (left) and inside the scanner (right) averaged over the three sessions of one representative subject. The frequencies on top of each plot correspond to the reversing frequencies of the visual flicker stimuli.

After this step, we computed Steady State Visual Evoked Responses (SSVERs) to further assess the goodness of the MR-related artefact correction method. These SSVERs were computed by first epoching the artefact-corrected 29-electrode EEG data acquired inside the MRI scanner, for each subject/session, in half-second (500 ms) post-stimulus window and then averaging across trials. Spectral analysis was then performed on the epoched and averaged EEG, using the data from electrode O2 (8 averaged epoch time series corresponding to the different stimuli used). The time-frequency spectra were constructed using Wavelets, as previously described in the Materials and methods section (Eqs. 1 and 2). The same procedure was then performed to obtain the SSVERs for the EEG data acquired outside the MRI scanner with the same experimental conditions, including the same paradigm. The responses obtained for both datasets were then compared. Fig. 3 shows the averaged SSVERs over all sessions of one representative subject for different frequencies of visual flicker.

As can be seen in Fig. 3, the major component of the spectra is at the second harmonic of the stimulus frequency. This result was expected since for reversing stimuli the SSVERs are usually produced at the phase-reversal or alternation frequency, which is twice the stimulation frequency (Burkitt et al., 2000). This fact also explains why almost no response is seen for the 30-Hz stimulus, for the range of frequencies here analysed (1 to 40 Hz).

However, for the purpose of this work, we were only interested in the similarity between the responses obtained inside and outside the scanner and as can be seen in Fig. 3 the close correspondence indicates that the MRI artefacts can be removed without filtering out the signal of interest.

The SSVERs were not used in the subsequent regression analysis. To compare the different transfer functions, we used the raw (artefact corrected, un-averaged and projected onto its first principal component) EEG data.

### Effect of the experimental task

We then looked at the effects of visual flicker on both the EEG and fMRI data. For the EEG data, the SSVER spectra shown in Fig. 3 provide evidence that visual cortical neurons synchronized their firing to the stimuli, leading to strong EEG responses at the second harmonic of the stimulus frequency.

**Fig. 4:**
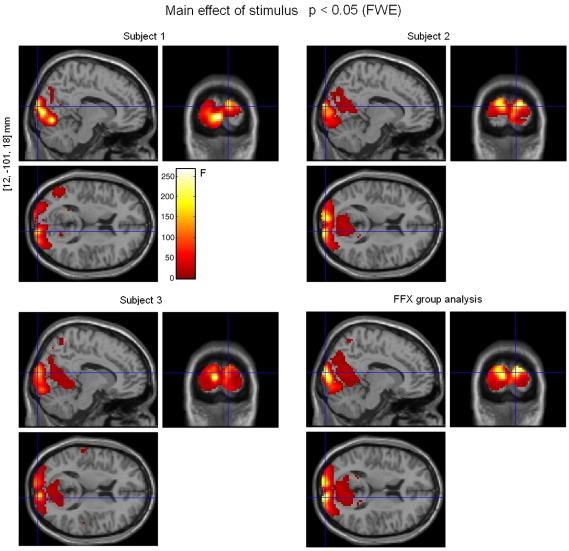
Effect of visual flicker stimulation on fMRI data. Single-subject analyses (3 sessions per subject) and fixed-effects group analysis (9 sessions in total), *p* < 0.05 (FWE). The voxel locations on the left correspond to the most significant cluster maximum for the group analysis (Talairach space).

For the fMRI data, both single subject and fixed-effects group analyses showed significant bilateral activation (*p* < 0.05 (FWE)) in visual areas of the occipital cortex (Fig. 4). These areas were identified with the help of the ‘Anatomy Toolbox’ for SPM software (Eickhoff et al., 2005). Talairach coordinates of cluster maxima [*x*,*y*,*z*] mm: right cuneus [12, − 101, 18], left superior occipital gyrus [− 9, − 101, 15], and right calcarine gyrus [3, − 92, 3] (Table 2). The fMRI images from the group analysis in Fig. 4 were used to create the BOLD activation mask, so that subsequent analyses could be restricted to BOLD activated regions.

In a separate analysis (not shown) which controlled for variation in luminance levels using an additional regressor of no interest, BOLD activity was shown to have an inverted U-shaped response to flicker frequency. The peak response was for a flicker frequency of 7.5 Hz and dropped off sharply above 15 Hz, agreeing closely with previous studies Singh et al. (2003), Parkes et al. (2004), and Wan et al. (2006). This result also explains why the amplitude of the SSVERs plotted in Fig. 3 decreases with increasing stimulus frequency, for both the responses obtained inside and outside the scanner (Fig. 3).

### Relationship between neuronal activity and BOLD

**Fig. 5:**
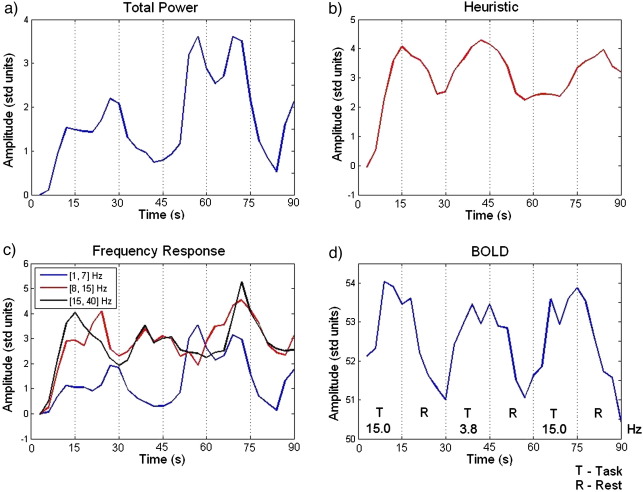
Example regressors for (a) Total Power, (b) Heuristic, and (c) Frequency Response (3 bands) models after convolution with the HRF (subject 2). (d) Example BOLD time series for the same period of time and subject, at the most significant cluster maximum ([12, − 101, 18] mm, Talairach space) from the fixed-effects group analysis of the main effects of visual stimulation (Fig. 4).

Fig. 5 plots example regressors for the Total Power, Heuristic, and Frequency Response (3 bands) models derived from Eqs. 3, 4, and 5, convolved with the Hemodynamic Response Function and downsampled to the fMRI frequency of acquisition. Fig. 5d plots an example BOLD time series for the same time interval and subject, at the most significant cluster maximum from Fig. 4 (fixed-effects group analysis), in relation to the paradigm. As can be seen there is an increase in BOLD during the ‘Task’ blocks which is better reflected in the Heuristic than in the other models. The highest frequency band of the Frequency Response model (Fig. 5c, black) also seems to follow BOLD more closely than the time series from the other bands.

**Fig. 6:**
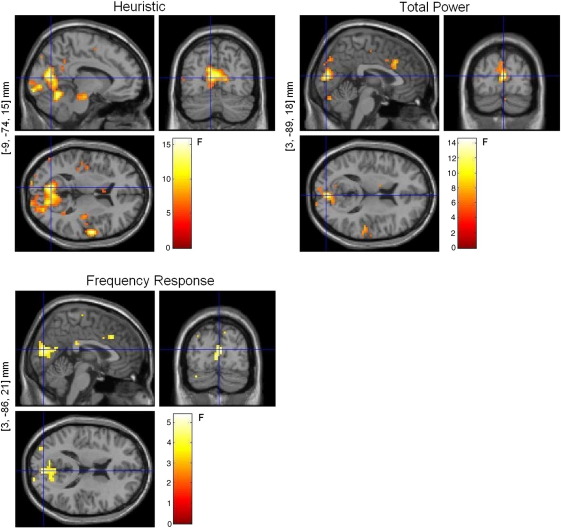
Fixed-effects SPM analyses (*p* < 0.001 (uncorrected)) for the Heuristic, Total Power, and Frequency Response (3 bands) models analysed in separate design matrices. The voxel locations on the left correspond to the most significant cluster maximum after small volume correction with the BOLD activation mask (Talairach space).

The SPM analyses with the separate design matrices (one for each model) showed significant (*p* < 0.05 (FWE)) correlations between each model and the observed BOLD signal, as can be seen in Fig. 6. The locations of maximal correlation for each model were not far apart and were included in the voxels activated by the experimental task shown in Fig. 4. Although all functions correlated with BOLD, the Heuristic produced higher maximal *F*-scores and more voxels above the chosen threshold (*p* < 0.05 (FWE)) than the other two models (Fig. 6).

**Fig. 7:**
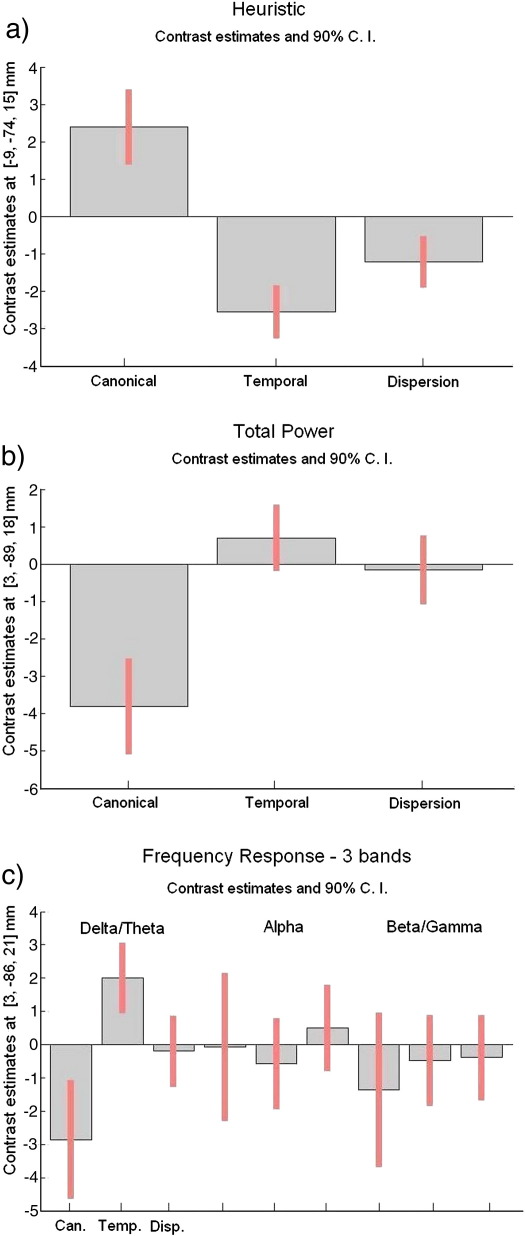
Contrast estimates and 90% CI for (a) Heuristic, (b) Total Power, and (c) Frequency Response with 3 bands (analysed individually). The estimates include the canonical HRF, as well as its temporal and dispersion derivatives.

The contrast estimates for the most significant voxel for each model showed that the Heuristic correlates positively with the amplitude of the BOLD response, while Total Power and the first frequency band of the 3-band Frequency Response model correlated negatively with this response (Fig. 7). Other sites showed significant correlation between BOLD and the other two frequency bands (not shown).

**Fig. 8:**
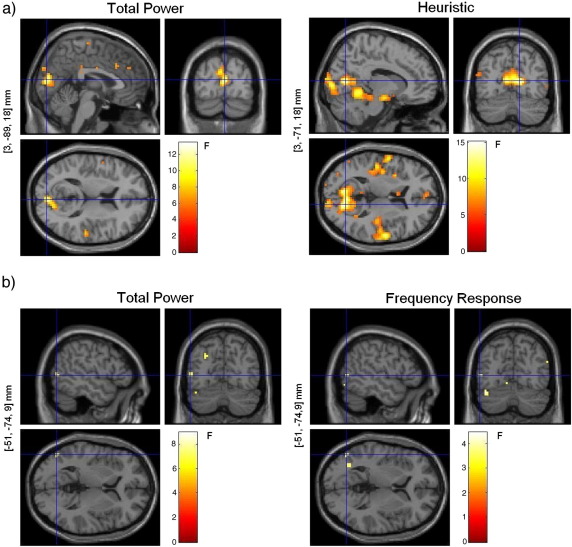
Two-way model comparison between (a) Total Power versus Heuristic and (b) Total Power versus Frequency Response (fixed-effects SPM analyses (*p* < 0.001 (uncorrected)). The voxel locations on the left correspond to the most significant cluster maximum after small volume correction with the BOLD activation mask (Talairach space). These *F*-maps show the correlations between EEG and BOLD that are uniquely attributable to each model within a pair.

We then performed two pairwise comparisons (a) between Total Power and Heuristic and (b) between Total Power and the Frequency Response model (Fig. 8). We included the regressors for the two functions, we were interested in comparing in the same design matrix. The results clearly revealed that the Heuristic provides a much better fit to the data than Total Power. For the second comparison it was difficult to see the effects of each model, since the regressors for the Total Power and particularly the firstband from the Frequency Response function (3 bands) were highly correlated.

**Fig. 9:**
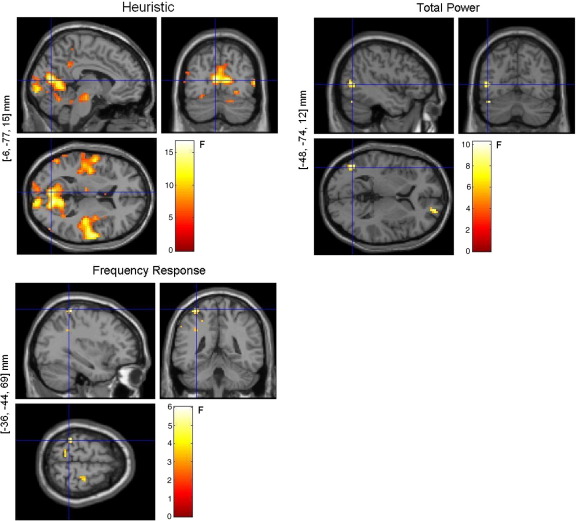
Three-way model comparison: fixed-effects SPM analyses (*p* < 0.001 (uncorrected)). Heuristic, Total Power, and Frequency Response (3 bands). The voxel locations on the left correspond to the most significant cluster maximum after small volume correction with the BOLD activation mask (Talairach space). These *F*-maps show correlations between EEG and BOLD that are uniquely attributable to each model.

The three-way comparison, using regressors from all models in the same design matrix, showed a much more widespread and stronger relationship between the Heuristic regressors and the BOLD signal compared to the Total Power or the Frequency Response functions, *p* < 0.001 (uncorrected) (Fig. 9). Furthermore, only the Heuristic showed significant correlations when we corrected for multiple comparisons, (*p* < 0.05 (FWE), using a small volume correction (SVC) over the bold activation mask), and the clusters that remained after SVC were located in the right and left calcarine gyrus (Talairach coordinates [*x*,*y*,*z*] mm: [3, − 92, 10] and [− 6, − 77, 15, respectively) and in the left cerebellum (Talairach coordinates [*x*,*y*,*z*]: [− 12, − 62, − 12] (Table 2).

These results are summarised in Table 3, where the number of voxels and the highest *F*-scores obtained for each model, within and outside the activation mask, for different thresholds can be found. The number of voxels, as well as the *F*-statistics, in both locations and thresholds were significantly higher for the Heuristic than for the other models (Table 3).

This three-way comparison is the main result of our paper and it was replicated in a case study analysis (Penny and Holmes, 2006) in which data from the different subjects were analysed separately. The individual results were very consistent across subjects: the Heuristic model was markedly superior for all three subjects (individual SPMs not shown), by producing higher *F*-scores than the rest of the models and more activated voxels inside and outside the brain activation mask. These results are summarised in Table 3 (individual tables not shown).

These results were also reproduced when we analysed 1800 scans instead of 900 (see above). Moreover, the inclusion of more data produced even higher statistics and more significant voxels (in the same brain areas reported) for the Heuristic than for the other models (not shown).

We also compared our three main models (Heuristic, Total Power, and Frequency Response (3 bands)) with the global field power of the EEG signal as described in the Materials and methods section. Therefore, we added this model to our fixed-effects design matrix. However, the inclusion of this function did not affect the previously obtained results (maps not shown), and the Heuristic again provided a better fit to the data, by producing more spatially distributed significant activations (*p* < 0.05, FWE corrected) and higher *F*-scores than the other models, including the GFP. These comparisons allowed us to reject the hypothesis that a model based purely on variations of amplitude across the EEG channels could provide a better fit to the BOLD data.

In addition, when we compared the Heuristic with the single-band Frequency Response models, the Heuristic also revealed more significant voxels and higher statistics than the Alpha and Beta/Gamma power. Moreover, inside the brain activation mask, the number of voxels where the Heuristic provided a better fit (FWE corrected) was 939 (maximum *F*-statistic: 23.9) when compared with Alpha, and 1480 (*F*_max_ = 31.8) when compared with Beta/Gamma. These two models showed only 69 (*F*_max_ = 16.8) and 293 (*F*_max_ = 20.9) activated voxels in this region, respectively. This result showed that reducing the number of bands in the Frequency Response model did not improve the performance of this model when compared to the Heuristic.

As an aside, we note that although the fMRI data were slice time corrected, significant variability was explained by the temporal derivative regressors (SPMs not shown), and therefore their inclusion in data analyses such as these are recommended (see for example Fig. 7a).

**Fig. 10:**
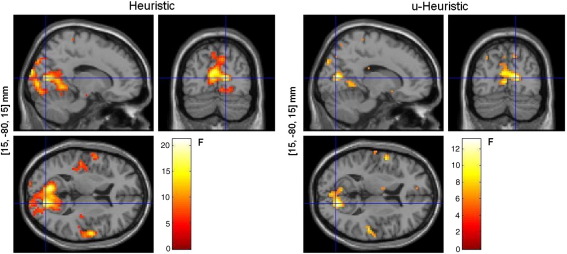
Comparison between Heuristic and its un-normalised version, the u-Heuristic: fixed-effects SPM analysis (*p* < 0.001 (uncorrected)). The voxel locations on the left correspond to the most significant cluster maximum after small volume correction with the BOLD activation mask (Talairach space). These *F*-maps show correlations between EEG and BOLD that are uniquely attributable to each model.

Comparing the Heuristic model and its un-normalised version, the u-Heuristic, revealed that the Heuristic significantly correlated (*p* < 0.05 (FWE)) with the observed BOLD data in most of the brain areas revealed when this function was compared to the Total Power and the Frequency Response models (Fig. 10). Applying the BOLD activation mask showed that the site with the most significant result was located again in the right calcarine gyrus (Talairach coordinates [*x*,*y*,*z*] mm: [15, − 80, 15], *p* = 1.71e− 09 (FWE), SVC) (Table 2). In this area, BOLD correlated positively with the Heuristic and negatively with u-Heuristic.

Finally we looked at the importance of the non-linearity present in the RMSF function for the Heuristic model, introduced by the square root operator (the R in RMSF). This was addressed by performing the following two-way model comparisons between (i) the Heuristic and its linear version, the l-Heuristic (Eq. 7); (ii) the Heuristic and the Frequency Response model but using normalised power (eight bands of 5 Hz each); and (iii) the Heuristic and the mean frequency function (Eq. 8). The rationale behind the second comparison is that the Frequency Response model based on normalised rather than un-normalised power should be able to implement the transfer function by assigning regression coefficients, *β*_*f*_ = *f*^2^. The results from these comparisons (SPMs not shown) were very similar. Although when analysed separately all these functions correlate significantly with the BOLD data at a high statistical threshold (*p* < 0.05 (FWE)), when put in the same design matrix none of the models is able to uniquely explain significant variation in BOLD. These results indicate that the non-linearity introduced by the square root function is not critical.

## Discussion

In this paper, we have used simultaneously acquired EEG and fMRI data, with a visual flicker stimulation task, to probe the transfer function from neuronal activity to BOLD. We compared three different models, each assuming BOLD is sensitive to a different feature of the EEG. These were (i) the Total Power model, (ii) the Frequency Response model, and (iii) the Heuristic model. When analysed in separate design matrices, all transfer functions correlated with the observed BOLD data, as expected.

For the Frequency Response model, all bands showed significant correlations with the data, in agreement with recent monkey EEG-fMRI results (Goense and Logothetis, 2008).

One initially surprising finding was that, at the location of maximal correlation, Total Power correlated negatively rather than positively with BOLD. However, this can be understood by noting that most of the power in the EEG signal, over rest and stimulus blocks, lies in the lower frequencies of the spectrum. This was confirmed by the negative correlation found in the lowest frequency band of the 3-band Frequency Response model in agreement with Mukamel et al. (2005) and Laufs et al. (2006a). Work in which positive correlation was observed, for example Wan et al. (2006), focussed rather on event-related power (rather than power in both rest and stimulus blocks). In addition, the fact that we modelled the relation between neuronal activity and BOLD in both stimulus and rest blocks together, implies that the Heuristic is also applicable to spontaneous neural activity.

The results of the two-way model comparison, between Total Power and the Heuristic, showed that the transfer function from neuronal activity to BOLD is frequency dependent. The three-way comparison was again clearly in favour of the Heuristic which was shown to explain significantly more BOLD activity than the other two models.

Independent of model, the majority of the voxels that were significantly correlated with the regressors were in the occipital cortex (Fig. 6). This is not surprising as we used flickering visual stimuli. What is perhaps surprising is that other brain areas outside of the occipital cortex (such as the cerebellum and temporal cortex) were also significantly correlated with some of the regressors, most notably for the Heuristic model (Fig. 9). It should be noted that as the Heuristic is a function of the power spectrum and is not a function of any one particular frequency, it may capture some dynamics that are not a simple entrainment of neural populations at some harmonic of the flicker rate.

One concern we had regarding the two and three-way comparison results was that the Heuristic may be better than the Frequency Response model simply because of the small number (three) of frequency bands used. However, our conclusions remained unchanged for frequency response models with additional numbers of bands (five and eight). Conversely, one might also think that the Frequency Response model could do better with a smaller number of frequency bands. The limiting case of this is a single frequency band. Two-way model comparisons, however, revealed the Heuristic to be better than using either (8–15 Hz) alpha or (15–40 Hz) high (beta/gamma) power alone.

Our attention then turned to what it is about the Heuristic that makes it a good model. We first addressed the issue of power normalisation. Comparison with a ‘scaled’ Heuristic, based on un-normalised rather than normalised spectra, revealed the original Heuristic to be clearly superior. The use of normalised power therefore seems important.

We then addressed the issue of non-linearity. This derives from the square root operator in Eq. 5 (the R in RMS). A direct comparison of the Heuristic with its linear version based on the MSF, as well as the Heuristic and the MF model, showed that when included together in the same design, the predictive power of both functions were reduced by the other. Similarly, model comparison of a normalised Frequency Response model with the Heuristic revealed that neither model showed superior predictive power. These results together indicate that, empirically, the non-linearity introduced by the square root function does not appear to be critical. A caveat however is that this conclusion may only be valid for the range of frequencies generated in this experiment (1 to 40 Hz).

A concern with the model comparison approach taken in this paper is that it is based on GLMs and *F*-tests, which restrict one to making inferences about nested models. If no natural nesting exists, then the regressors from all models are placed in the same design matrix and *F*-tests used to infer whether sets of variables explain additional variance. While this approach is commonplace (Friston et al., 2007), it is nonetheless suboptimal as compared to direct comparison of models using the Bayesian model evidence criterion (Penny et al., 2007). We have recently extended this Bayesian model comparison approach to data from group studies (Rosa et al., in press) and plan to apply it to our EEG-fMRI data.

A further concern in the analyses we have presented here is in the use of EEG regressors as a surrogate for neuronal activity. This approach has previously been used by a number of groups (Lemieux et al., 2001; Goldman et al., 2002; Laufs et al., 2003; Moosmann et al., 2003). In this paper, we followed the same rationale but additionally employed a visual flicker stimulation paradigm to elicit evoked activity in sensory cortex. We then used the first principal component of the EEG data to isolate activity that was primarily related to the stimulus paradigm. We note that this approach could be improved in a number of ways. First, one could employ multiple PCA or ICA components (Vigario et al., 2000; Eichele et al., 2005, 2009), which might better isolate activity from specific processes or brain regions. Second, one could use regressors derived from EEG source reconstructions as in Wan et al. (2006). A problem with these approaches, however, is that they are no longer compatible with a whole-brain SPM analysis approach, as that requires the same design matrix at all voxels. They are nevertheless worth pursuing and we hope to do so in future publications.

In the longer term, however, we envisage that such ‘asymmetric’ (Kilner et al., 2005) regression approaches will be superseded by ‘symmetric’ forward models, such as proposed in Sotero and Trujillo-Barreto (2008). Interestingly, this forward modelling approach based on neural mass models also supports the Heuristic, as exogenous input causes both a BOLD activation and an increase in the mean LFP frequency (Sotero and Trujillo-Barreto, 2008).

Some results in the literature may appear at odds with the Heuristic. For instance the positive correlations with alpha power found in the thalamus by Goldman et al. (2002) and in other regions (Gonçalves et al., 2006). However, the Heuristic describes a relationship based on normalised not absolute power. Therefore, if increases in alpha were, for example, accompanied by decreases in lower frequencies (delta/theta), this would be compatible with the Heuristic. Using separately acquired fMRI and source-reconstructed MEG data, Muthukumaraswamy and Singh (2008) showed stimulus-related increases in gamma band activity without corresponding changes in BOLD. However, while this result clearly speaks against the gamma-BOLD hypothesis, it does not necessarily speak against the Heuristic. This is again because the Heuristic depends on the normalised power of the whole spectrum.

An interesting inference to be drawn from Muthukumaraswamy and Singh (2008) is that gamma band power may reflect the synchronized activity of *local* neuronal ensembles. This view fits in with neural network modelling results (Kopell et al., 2000) and power-law analyses of electrocorticogram data (Miller et al., 2007). While BOLD can be sensitive to changes in the gamma band, as many studies have shown, it is also sensitive to activity in the whole spectral domain, including the more spatially dispersed lower frequencies (Kopell et al., 2000), and processes reflecting large-scale neuromodulatory input (Logothetis, 2008).

The original paper that described the Heuristic model was partly inspired by the results of EEG-fMRI integration in the study of epilepsy. In this field, increased slow wave activity has been shown to be associated with decreased BOLD (Archer et al., 2003) while spike and wave discharges (with high-frequency components) have been shown to cause BOLD activations (Krakow et al., 2001; Hamandi et al., 2004). This would be entirely in agreement with the Heuristic model.

To our knowledge, this paper reports the first study where the model proposed by Kilner et al. (2005) has been empirically tested using human brain imaging data. It is also the first work in which different putative functions for the relationship between BOLD and spectral characteristics of neuronal activity, as measured with EEG, have been explicitly compared.

To this end we designed a study providing experimental control over the frequency structure of the EEG signal by entraining networks to visual stimulation at different frequencies. Our results suggest that changes in BOLD are indeed associated with changes in the spectral profile of the underlying neuronal activity, and that these changes do not arise from a single spectral band. Instead they result from the dynamics of the various frequency components together, in particular, the relative contribution of high and low frequencies as proposed in Kilner et al. (2005).

Although we entrained networks to visual stimulation, we have no reason to anticipate different results if neuronal activity were modulated by different cognitive processes. However, this is an empirical question that should be addressed in future studies. The current paper provides evidence in favour of the Heuristic model but, of course, as with any scientific experiment does not prove that the underlying theory is true. We expect that as data are gathered from additional experimental paradigms and sensory modalities, a balance of evidence will emerge.

We expect that fMRI recorded concurrently with intra-cranial EEG will play a major role in these investigations as this will provide more direct access to the various cortical and subcortical regions that have little impact on the scalp EEG. This may help to resolve to what extent, if at all, BOLD and EEG are differentially sensitive to endogenous lower frequency ‘global’ states versus higher frequency local processing (Laufs, 2008).

Understanding the nature of the link between neuronal activity and BOLD plays a crucial role in improving the interpretability of BOLD imaging and relating electrical and hemodynamic measures of human brain function. Finding the optimal transfer function should also aid the design of more robust and realistic models for the integration of EEG and fMRI, leading to estimates of neuronal activity with higher spatial and temporal resolution, than are currently available.

## Figures and Tables

**Table 1:** Summary of model comparisons and corresponding number of estimated parameters.

Model comparisons		*n*_R _× *n*_BF _+ *n*_C _= *n*_P_	*n*_*P*_ (9 sessions)
i.	Heuristic	1 × 3 + 7 = 10	90
Total power (TP)	1 × 3 + 7 = 10	90
Frequency response 3 bands (FR^3^)	3 × 3 + 7 = 16	144
ii.	TP vs. Heuristic	(1 + 1) × 3 + 7 = 13	117
TP vs. FR^3^	(1 + 3) × 3 + 7 = 19	171
iii.	TP vs. FR^3^ vs. Heuristic	(1 + 3 + 1) × 3 + 7 = 22	198
iv.	TP vs. FR^3^ vs. Heuristic vs. GFP	(1 + 3 + 1+ 1) × 3 + 7 = 25	225
v.	Heuristic vs. FR^1^	(1 + 1) × 3 + 7 = 13	117
vi.	Heuristic vs. u/l-Heuristic/MF	(1 + 1) × 3 + 7 = 13	117
Heuristic vs. FR^5^/FR^8^	(1 + 5/8) × 3 + 7 = 25/34	225/306

For one session: *n*_R_ is the number of regressors of interest for each transfer functions; *n*_BF_ is the number of basis functions, which is always 3 (canonical HRF, temporal, and dispersion derivative); *n*_C_ is always 7 and corresponds to the number of confounds (6 motion parameters and 1 mean regressor); and *n*_P_ is the total number of parameters to be estimated for each comparison.

**Table 2:** Anatomical location in Talairach space of the sites with significant results from the three-way model comparison (fixed-effects SPM analysis, without SVC).

Regressors	[*x*,*y*,*z*] (mm)	Location	Inference
Stimuli	[12, − 101, 18]	Right cuneus	*p* < 0.05 (FWE)
[− 9, -101, 15]	Left Superior Occipital Gyrus	
[3, − 92, 3]	Right calcarine gyrus	
Heuristic	[− 6, − 77, 15]	Left Calcarine Gyrus	*p* < 0.05 (FWE)
[3, − 92, 10]	Right calcarine gyrus	
[− 54, − 17, 9]	Left Superior Temporal Gyrus	
[60, − 11, 15]	Right Rolandic Operculum	
[− 12, − 62, − 12]	Left Cerebellum	
Total power	[− 48, − 74, 12]	Left Middle Temporal Gyrus	*p* < 0.001 (uncorrected)
Frequency response	[− 48, − 74, 12]	Left Middle Temporal Gyrus	*p* < 0.001 (uncorrected)
[− 42, − 74, − 15]	Left Cerebellum	

**Table 3:** Summary of results for the three-way comparison between Total Power (TP), the Heuristic, and the 3-band Frequency Response (FR) models from the fixed-effects group analysis (Fig. 9).

Location	Threshold	*n*_vox_|*F*_max_		
Heuristic	TP	FR^3^
Within BAM				
	*p* < 0.05 (FWE)	17|13.3	0|–	0|–
*p* < 0.001 (uncorrected)	620|13.3	5|8.2	18|4.9
Outside BAM				
	*p* < 0.05 (FWE)	7|13.3	0|–	0|–
*p* < 0.001 (uncorrected)	801|13.3	46|9.6	95|4.8

‘BAM’ is the brain activation mask obtained from the main effects of stimulation (Fig. 4); *n*_vox_ is the total number of voxels within a certain area; and *F*_max_ the maximum *F*-statistic within that region.
